# Mesocosm experiment reveals scale dependence of movement tendencies in sticklebacks

**DOI:** 10.1098/rsbl.2022.0602

**Published:** 2023-04-05

**Authors:** A. Ramesh, J. Gismann, T. G. G. Groothuis, F. J. Weissing, M. Nicolaus

**Affiliations:** Groningen Institute for Evolutionary Life Sciences, University of Groningen, Nijenborgh 7, 9747 AG Groningen, The Netherlands

**Keywords:** animal behaviour, migration, population divergence, passive integrated transponder (PIT) tag, radio-frequency identification (RFID)

## Abstract

Habitat fragmentation can have negative impacts on migratory organisms that rely on the functional connectivity between growing and breeding grounds. Quantifying the population-level phenotypic consequences of such fragmentation requires fine-scaled tracking of individual behaviour and movements across relevant scales. Here we make use of a natural experiment where some populations of ‘migrant’ three-spined sticklebacks (*Gasterosteus aculeatus*) became ‘residents', following habitat fragmentation five decades ago. To test whether residents have a lower movement tendency than migrants, we developed a novel experimental platform that allows the automated tracking of individual movements via RFID technology in a semi-natural mesocosm where spatio-temporal scales and environmental conditions can be manipulated. We found that residents moved significantly less than migrants at large but not at small spatial scale. This pattern was consistent across time and contexts (water flow and group size). Our study substantiates prior literature on rapid phenotypic divergence in sticklebacks in response to human-induced isolation and highlights the importance of observing behaviour in ecologically relevant set-ups that bridge the gap between laboratory and field studies.

## 1. Introduction

Habitat fragmentation is a major threat for many animals, particularly for migratory species that depend on multiple habitats to complete their life cycle [1]. Water management efforts worldwide have disrupted the connectivity between marine and freshwater habitats, confining some fish populations to only freshwater habitats without the possibility of migrating to the sea. Such forced isolation can cause rapid phenotypic responses and life-history changes (mammals and birds: [2]; fish: [3–6]). Species' responses to reduced connectivity, forced isolation, decreased densities and smaller population sizes will inform us whether and how animal populations can cope with these human-induced changes. In this context, behavioural responses are crucial in determining success in persisting in fast-changing environments, especially in the initial stages [7]. However, studying such responses typically requires quantification of behaviour and movement at the individual level, which is often challenging in small-bodied species and especially so in the aquatic environment. For example, in the wild, individual tracking of small organisms’ movements is often either impossible or gives rise to uncertainty in the observations, as a large proportion of data is often missed (mark–recapture experiments in the wild often have low recapture rates or biased recapture due to animal behaviour or weather conditions [8,9]). While laboratory studies can be highly controlled and allow for experimental manipulation, they regularly suffer from spatio-temporal limitations and most importantly lack ecological relevance due to limited environmental complexity compared to natural environments [10–13].

Using individual laboratory-based assays, we have previously shown that, in the Netherlands, ‘resident’ populations of three-spined sticklebacks (*Gasterosteus aculeatus*), isolated in freshwater ditches due to man-made barriers for approximately 50 years, diverged in behaviour (and morphology) from their ‘migrant’ ancestors [14]. Yet, contrary to our expectations (and other findings for example on higher swimming performance and endurance of migrants in comparison to residents [15,16]), migrants did not exhibit higher movement tendencies than residents. Moreover, residents were more active and exploratory than migrants [14]. We speculated that the counterintuitive nature of the results could be due to a freezing or stress response of migrants in the absence of a social group [14,17]. Further, the small-scale experimental settings in the laboratory may not be suited to study larger scale movement, which we explicitly aim to test here.

In recent decades, the use of passive integrated transponders (PIT tags) has become very prominent in the studies of movement patterns in wild populations [18], including small organisms such as passerine birds [19,20], insects [21] and fish [22]. With this technology, individual behaviour and social associations can be measured using remote detections at fixed locations. In this study, we describe a novel experimental mesocosm set-up that uses PIT tags and a radio-frequency identification (RFID) system to quantify individual behaviour and movement of small fish in semi-natural conditions, thus providing relevant environmental complexity and temporal scales while allowing the investigation of movement across different spatial scales. The mesocosm consists of several connected semi-natural ponds equipped with RFID antennas to monitor individual movement tendencies. Here we particularly address if (i) spatial scale matters for uncovering population divergence in movement tendencies and (ii) movement tendencies of migrant and resident fish are consistent across ecological conditions such as water flow and group size.

## 2. Methods

### (a) Mesocosm system

**Figure 1.**
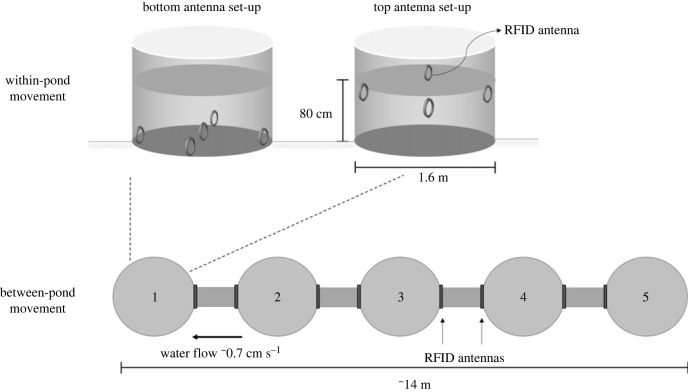
Experimental set-up. Each mesocosm consisted of five linearly connected above-ground ponds (1–5) equipped with circular RFID antennas that automatically detect crosses of PIT-tagged individuals. Fish were released into pond 1. This pond was equipped with nine RFID antennas (five on the bottom and four on top of the water column), allowing us to quantify within-pond movements. The connections between adjacent ponds were equipped with two RFID antennas, allowing us to quantify the number and direction of movements between ponds.

The experiments were conducted in two independent mesocosms of five above-ground ponds (each Ø 1.6 m, with a water depth of 80 cm), connected linearly with opaque corridors (each of length approx. 1.5 m and Ø 11 cm), spanning a linear distance of approximately 14 m (figure 1). The system is supplied with freshwater from a natural ditch with the possibility of creating water flow (approx. 0.7 cm s^−1^) that mimics conditions in Dutch canals and represents a directional migration cue together with seasonal changes of temperature and photoperiod [23]. This system enabled measurement of the movements of individual sticklebacks within- and between-ponds. The first pond (labelled 1 in figure 1), enriched with plastic plants, was used to quantify within-pond movement, while the whole system of five connected ponds was used to record between-pond movement tendencies (see electronic supplementary material, S1 for details).

Our tracking system consisted of circular RFID antennas (Ø 10 cm), data loggers and PIT tags (Trovan, Ltd, Santa Barbara, California) to record movements of PIT-tagged sticklebacks (details in electronic supplementary material, S2). Nine circular antennas were placed in the first pond to record within-pond movements, and two antennas were placed at both ends of each of the four connecting corridors to measure between-pond movement (figure 1). Each antenna records the unique PIT tag ID of the fish along with a time stamp, stored on a USB drive in the central data logger. The sensitivity of the system was set to three reads per second per unique tag. In a pilot study, we validated the reads using video recordings and found that no detections were missed and that reads corresponded well with the entry and exit times of fish.

### (b) Experiment 1: movement across spatial scales in the mesocosm

We created five groups of migrants and six groups of residents, each consisting of 10 randomly selected individuals (total: *Nmig* = 49 and *Nres* = 60). While we always tried to maintain the group size to 10 fish by making up the group to 10 with untagged individuals, tag loss and other technical difficulties (such as retagging and waiting for recovery) led to one group of migrants having nine fish and another with 11 fish, in order to test all the tagged fish. Groups were housed in separate small holding ponds for 24 h before the start of the experiment. On the experimental day, one resident and one migrant group were released simultaneously (to avoid temperature or temporal biases) into separate mesocosms. The individuals in each group were first monitored for within-pond movement by confining the fish to the starting pond for the first 5 h (figure 1) and then for between-pond movement for approximately 16.5 h, after opening the connection to the other ponds (figure 1; electronic supplementary material, S1 and S2).

### (c) Experiment 2: effect of group size and water flow on movement

In a next step (after about one month), we combined all migrants and, separately, all residents (after excluding 12 fish that had either died or lost tags) into two large groups, which reflect natural conditions more closely, as stickleback prefer larger over smaller groups [24] (*Nmig* = 45, *Nres* = 52; one group of migrants and one group of residents) and quantified between-pond movements in these two groups in the two mesocosm set-ups over 4 days. In addition, we alternated flow and no-flow conditions on consecutive days (see electronic supplementary material, S1).

### (d) Analyses

We first cleaned the data from small read errors which could be easily corrected given the high sensitivity of the RFID system (3–5 reads/second). Then, for each individual, we quantified within-pond movement as the number of times each fish moved between different bottom antennas or different surface antennas, respectively (figure 1). We deemed the number of consecutive visits to a particular antenna unreliable for measuring movement patterns because of the possibility that a prolonged visit to a given antenna might be recorded as multiple disconnected set of reads, appearing as if the fish visited the antenna multiple times. Between-pond movement was quantified as the number of crosses a fish made through the corridors connecting two ponds (figure 1). Fish that were not detected by any antenna were given a score of zero crosses.

We then analysed if residents and migrants differed in the number of crosses for within- and between-pond movements and whether they were consistent across contexts (group size and flow). Briefly, we considered the number of crosses within- and between-ponds as response variables separately in univariate generalized linear mixed models with Poisson errors. In all models, we included *origin* (resident versus migrant) as a fixed factor and *group-ID* (to account for pseudo replication) and an observation level *‘Obs’* (to control for overdispersion, [25]) as random effects. Additionally, we analysed whether the fraction of fish that did not exit the first pond differed between migrants and residents in Experiment 1, using Fisher's exact test. Repeatability and correlation of number of crosses across contexts were also calculated (electronic supplementary material, S3). For Experiment 2 (effect of group size and water flow on movement), our effective sample size was one per origin, hence we refrained from conducting statistical analysis and interpreted results from the plot. We estimated individual consistency of between-pond movements using repeatability and correlation coefficients, which are provided in the electronic supplementary materials (electronic supplementary material, S3). All analyses were carried out in R v. 4.1.0, [26]. Complete description of the analyses and code are given in the electronic supplementary materials.

## 3. Results

### (a) Movement across spatial scales in the mesocosm

**Figure 2.**
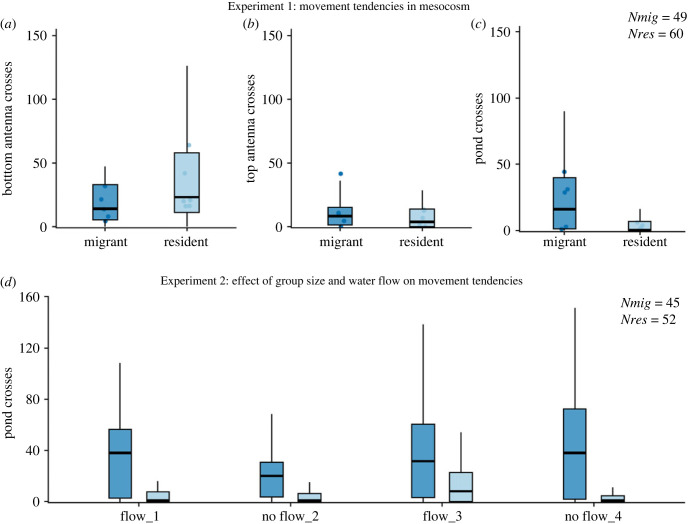
Within-pond and between-pond movement of resident and migrant sticklebacks. (*a*,*b*) Within-pond movement between bottom and top antennas, respectively (Experiment 1); (*c*) between-pond movement in Experiment 1; (*d*) between-pond crosses in relation to the daily flow treatment in Experiment 2. In all graphs, boxplots with median are shown for migrant (dark blue) and resident (light blue) sticklebacks. For (*a–c*), we have also represented the medians of each test group as shaded dots (migrant = five groups, resident = six groups).

Within the first pond, residents and migrants showed a broad distribution of number of crosses at both bottom and top antennas (figure 2*a,b*) and the number of crosses were not different between the two groups in both cases (table 1; median bottom-antenna crosses: residents = 23, migrants = 14; median top-antenna crosses: residents = 3.5, migrants = 8). By contrast, the number of movements between ponds was smaller in residents than in migrants (figure 2*c*; effect of *Origin* in table 1; median pond crosses: residents = 0, migrants = 16). Furthermore, the proportion of ‘non-leavers’, i.e. individuals that did not exit the first pond, was higher in residents than in migrants (55% in residents versus 28.6% in migrants, odds ratio = 3.02, *p* = 0.007).

**Table 1.** Results of the statistical analysis of movement within- and between-ponds using generalized linear mixed models. Estimates of fixed effects (*β*) on a log-scale are given with their 95% confidence intervals (CI), *Z*-values (Wald statistic), *p*-values and variance components are given with their s.d. Sample sizes Experiment 1: *Nmig* = five groups (49 individuals), *Nres* = six groups (60 individuals).

	Experiment 1: movement across spatial scales											
	bottom crosses				top crosses				pond crosses			
fixed effects	*Β*	95% CI	*Z*-value	*p*	*β*	95% CI	*Z*-value	*p*	*β*	95% CI	Z –value	*p*
intercept	2.61	(2.13, 3.08)	11.78	<0.001	0.90	(−1.66, 2.99)	1.00	0.31	1.90	(0.63, 3.13)	3.30	<0.001
origin^a^	0.51	(−0.12, 1.15)	1.72	0.09	−0.35	(−0.87, 0.16)	−1.36	0.17	−2.26	(−4.04, −0.58)	−2.82	0.005
random effects	Var (s.d.)				Var (s.d.)				Var (s.d.)			
group-ID	0.11 (0.33)				4.44 (2.11)				0.95 (0.98)			
Obs	1.21 (1.10)				1.29 (1.13)				5.02 (2.24)			

^a^‘migrant’ is used as reference category.

### (b) Effect of group size and water flow on movement

In large social groups, residents again moved consistently less between ponds than migrants (median pond crosses over the 4 experimental days range between 1 and 6 for residents and between 20 and 38 for migrants, figure 2*d*). Therefore, we conclude that differences between residents and migrants were maintained regardless of changing group size or differing ecological (flow) conditions.

## 4. Discussion

Previous studies in sticklebacks that have quantified population movement tendencies under laboratory conditions showed mixed or counterintuitive patterns: residents showed either higher [14] or inconsistent patterns [27] in activity/exploration levels compared to migrants. In this study, we show that migrants and residents differ in their movement tendency only on a larger spatial scale (between-ponds), while no differences could be detected on a smaller scale (within-ponds). It is thus conceivable that previous inconsistencies stem from the fact that the experimental set-ups did not offer biologically relevant testing conditions. The use of semi-natural mesocosms, as described here, may thus be a more appropriate way to characterize individual and population movement related to migration.

It is biologically plausible that movement tendencies are scale dependent. Movement measured at very different spatial scales (from 30 cm in the laboratory to 1.5 m within-ponds to 14 m across ponds) may reflect functionally different behaviours. For example, individual measurements on smaller scales, in the laboratory, may be an indication of a stress response to social isolation [17]. By contrast, in the wild, sticklebacks exhibit considerable foraging movements over days (median of 40 m upstream, [28]) and, hence their within-pond movements, representing foraging movements, may not differ between populations. However, wild migrants in our field system travel tens of kilometres inland within a few days (pers. comm. from water authorities) and thus require sufficient space to express their natural behaviour.

Tests in the laboratory, though invaluable for studies on animal behaviour owing to controlled settings, are not without drawbacks. Laboratory tests are usually performed in highly controlled and novel environments. This can lead to homogenization of behavioural expression (e.g. decreased variance over time [29]) or uncovering ‘cryptic’ behavioural variation (with novel behaviours and increased variance in behavioural expression [30]). We thus advocate using mesocosms or other semi-natural set-ups (e.g. [21,31–37]), to bridge laboratory and field studies. They circumvent the mentioned drawbacks and may provide valuable insights undetectable in classical behavioural set-ups, especially for wild populations. In addition, the modular nature of the described mesocosm offers flexibility in the spatial organization of the individual ponds and antennas. This allows for classical tests, such as choice tests, to be conducted in a more sophisticated manner. We are confident that such systems, enabling remote tracking and yielding high-resolution data over longer periods of time will become common in behavioural studies.

Our results show that freshwater-induced phenotypic changes in sticklebacks can occur on contemporary timescales (see also [15,38,39]) and a follow-up study showed that some of these have a genetic component [40]. The direction of these phenotypic changes is similar to the behavioural and morphological adaptions reported in stickleback populations that have colonized freshwater habitats after the last glacial retreat [41–45]. Residents in our study populations are thus likely on a trajectory to lose their migration tendencies and already (partially) adapted to complete residency.

## Data Availability

Datasets and code for processing and analysing data are available from the Dryad Digital Repository: https://doi.org/10.5061/dryad.xwdbrv1hx [46]. Supplementary material is available online [47].
